# A Systematic Review of T Cell Epitopes Defined from the Proteome of Hepatitis B Virus

**DOI:** 10.3390/vaccines10020257

**Published:** 2022-02-08

**Authors:** Yandan Wu, Yan Ding, Chuanlai Shen

**Affiliations:** Department of Microbiology and Immunology, Medical School of Southeast University, Nanjing 210009, China; 220203902@seu.edu.cn (Y.W.); 230189322@seu.edu.cn (Y.D.)

**Keywords:** hepatitis B virus, T cell epitope, HLA restriction

## Abstract

Hepatitis B virus (HBV) infection remains a worldwide health problem and no eradicative therapy is currently available. Host T cell immune responses have crucial influences on the outcome of HBV infection, however the development of therapeutic vaccines, T cell therapies and the clinical evaluation of HBV-specific T cell responses are hampered markedly by the lack of validated T cell epitopes. This review presented a map of T cell epitopes functionally validated from HBV antigens during the past 33 years; the human leukocyte antigen (HLA) supertypes to present these epitopes, and the methods to screen and identify T cell epitopes. To the best of our knowledge, a total of 205 CD8^+^ T cell epitopes and 79 CD4^+^ T cell epitopes have been defined from HBV antigens by cellular functional experiments thus far, but most are restricted to several common HLA supertypes, such as HLA-A0201, A2402, B0702, DR04, and DR12 molecules. Therefore, the currently defined T cell epitope repertoire cannot cover the major populations with HLA diversity in an indicated geographic region. More researches are needed to dissect a more comprehensive map of T cell epitopes, which covers overall HBV proteome and global patients.

## 1. Introduction

Hepatitis B virus (HBV) infection still disseminates across the world and causes the most common and fatal liver diseases including acute liver failure, chronic hepatitis, liver cirrhosis (LC), and hepatocellular carcinoma (HCC) [1,2]. Nucleoside analogs and/or interferon are widely utilized antiviral drugs, which can effectively suppress virus replication, decrease serum HBV DNA to undetectable levels, mitigate liver fibrosis, and reduce HCC risk [3,4,5], however cannot eliminate the virus in patients. Recurrence after therapy discontinuation is emerging to be a common etiology of morbidity and mortality in patients with chronic HBV infection [6].

Numerous researches have demonstrated the important influence of HBV-specific T cell responses on virus clearance [7], disease progression [8,9,10], antiviral efficacy [11,12], and recurrence [13,14,15], particularly the CD8^+^ T cells, which act as vital effector cells to kill virus-infected hepatocytes and secret cytokines. Patients with acute-resolving HBV infection show robust HBV-specific CD8^+^ T cell responses, while the patients with chronic HBV infection present a phenomenon termed CD8^+^ T cell functional exhaustion with multifactorial heterogeneity [9], and differs depending on the targeted antigen for HLA-A02 restricted epitopes located in the core antigen versus polymerase [16]. Furthermore, the heterogeneity of HBV-specific T cells also responds differently to therapeutic stimuli [17]. Therefore, T cells specific for HBV not only are the potential markers for monitoring the effects of antiviral therapy and predicting the recurrence [18], but also are the promising modulators in specific immunotherapy. Identifying the T cell epitopes as many as possible from HBV antigens will greatly contribute to the design and development of epitope-based and T cell-based therapies and the detection of host HBV-specific T cell immunity. Although a systematic review of T cell epitopes in HBV antigens was reported in 2008 [19], an updated map of the T cell epitopes is urgently needed. 

Here, this review comprehensively collected the CD8^+^ T cell epitopes and CD4^+^ T cell epitopes defined from HBV proteome during the past 33 years. Information resources are the English language journals collected in Pubmed, Scopus, Embase, SinoMed, and Google Scholar databases. The latest online search was conducted on October 8, 2021. “T cell epitopes” and “HBV or hepatitis B virus” were used as specific searching terms. An initial search identified 451 studies from multiple databases and manual searches. All articles were imported to Endnote software X8 (Thompson and Reuters, Philadelphia, PA, USA) and 121 duplicates were removed. In total, 330 studies from 1988 to 2021 were collected. Then, 233 articles were filtered out after abstract and full-text screening, according to the exclusion criteria below: (1) not related to the screening or identification of T cell epitopes; (2) just using in silico prediction or molecular structure bioinformatic analysis rather than satisfactory cell functional experiments, tetramer staining, binding assay, stabilization assay, or immunization; (3) with incomplete information regarding epitopes sequences. Finally, 97 articles were analyzed and referenced in this review.

## 2. Polymorphism of HLA Alleles and Association with HBV Infection 

Human leukocyte antigens (HLA) are coded by human major histocompatibility complex and have multiple important functions. In particular, they present antigenic peptides (T cell epitopes) in the form of peptide/HLA complex to T cell receptors onto specific T cells by which to initiate the adaptive immune response. HLA class I molecules (classically HLA-A, -B, and -C) are constitutively expressed onto almost all nucleated cells with distinct levels and present antigenic peptides to specific CD8^+^ T cells, while HLA class II molecules (classically HLA-DR, -DQ and -DP) are mainly expressed onto professional antigen-presenting cells (APCs, including monocytes, macrophages, dendritic cells and B cells) and present peptides to specific CD4^+^ T cells. In virus infection, HLA class I molecules expressed by virus-infected cells present the viral endogenous epitope peptides to specific CD8^+^ T cells, thus initiating the naive CD8^+^ T cells to activate, proliferate and differentiate to cytotoxic T lymphocytes (CTLs). The resulting CTLs mediate the cytolysis of virus-infected cells by Fas/FasL, TNF/TNFL, and perforin/granzyme pathways [20]. HLA class II molecules expressed by APCs present exogenous viral peptides to CD4^+^ helper T cells, thus eliciting the naive CD4^+^ T cells to differentiate into effective Th1 or Th2 cells. The former help virus-specific CD8^+^ T cells activation and the latter help virus-specific B cells differentiate to plasma cells and produce antibodies [21]. However, HLA molecules are highly polymorphic in the general population. As of October 2021, a total of 24,284 alleles have been described at HLA class I and class II loci in the global populations, including 6921 HLA-A, 8181 HLA-B, 6779 HLA-C, 3801 HLA-DRB1, 2033 HLA-DQB1, and 1862 HLA-DPB1 alleles, according to the International Immunogenetics Information Project/HLA database (IMGT; www.ebi.ac.uk/imgt/hla/stats.html, accessed on 11 November 2021). HLA allotypes are distinctive from individual to individual, and each HLA allotype presents distinctive antigenic peptides, thus leading to different immune patterns in different individuals against the same pathogen such as HBV [22,23]. Among the different ethnic populations in different geographic regions, the distribution of prevalent HLA alleles is markedly different. For an instance, 13 kinds of predominant HLA-A allotypes (each allotype has a gene frequency of >1% in Chinese herd) gather a total HLA-A allele frequency of around 95.5% in the Chinese population while 94%, 83%, 80%, 70% and 63% in Northeast Asia, Southeast Asia, Europe, South America, and North America populations, respectively (http://www.allelefrequencies.net, accessed on 11 November 2021).

Consequently, some alleles of HLA molecules have increasingly been linked to the occurrence of the indicated diseases, which are usually associated with abnormal immune function and genetic tendency [24,25]. Although the association of HLA alleles with HBV infection is not well clarified, a few studies have indicated that HLA-DRB1*13 and HLA-DRB1*07 are related to susceptibility to chronic HBV infection, and DRB1*15 is negatively related to persistence to chronic HBV infection in the populations of Africans [26], Europeans [27], Koreans [28] and Northwestern Chinese [29]. In addition, HLA-A*33 is closely associated with susceptibility to persisting HBV infection, and HLA-DRB1*13 is closely related to protection against persisting HBV infection in an Iranian population [30]. A*0301 and DRB1*1302 are relevant to viral clearance and B*08 is associated with viral persistence in Caucasians [31]. However, although the correlation between HBV infection and HLA alleles has been studied for several decades, in accordance with what we described in the above review, it often has conflicting results. These variations partly result from host HLA polymorphism in different races and regions [32,33]. Further studies should be explored in different regions to reduce the heterogeneity of results.

## 3. HBV Proteome and the Approaches Identifying T Cell Epitopes

**Figure 1:**
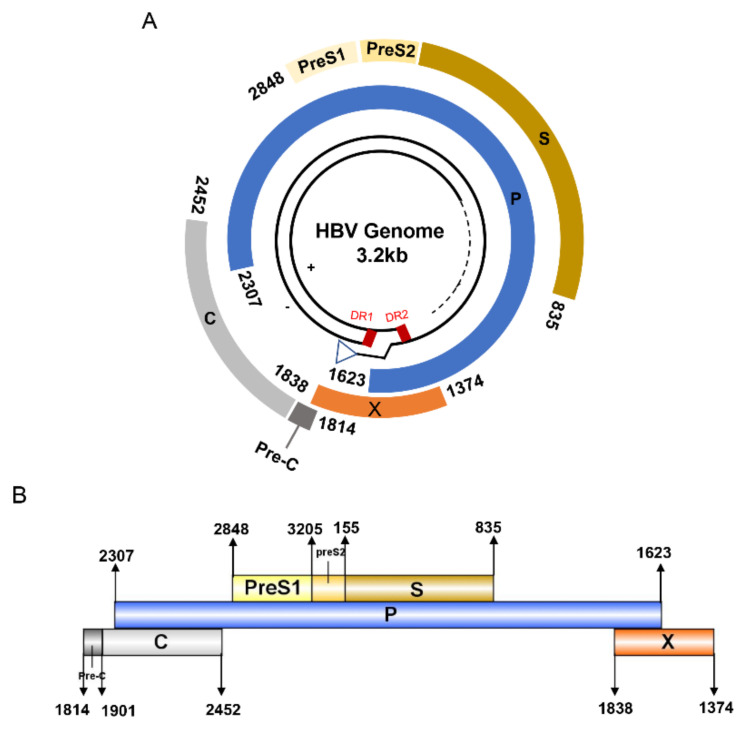
The circular (**A**) and linear (**B**) diagram of HBV genome.

**Figure 2:**
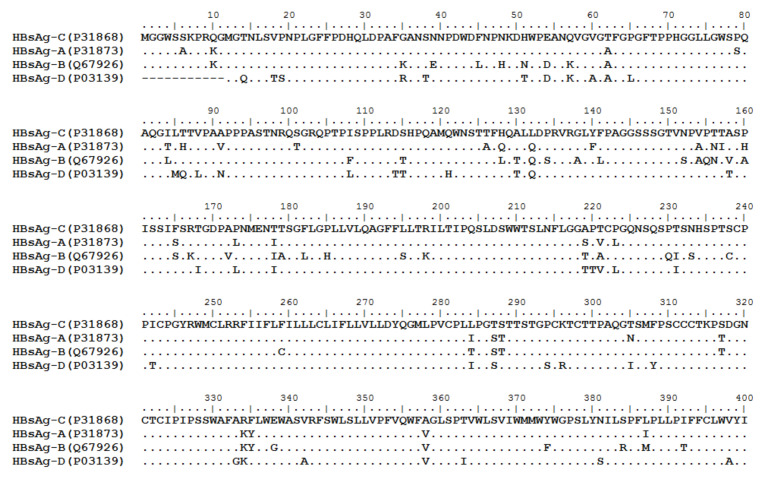

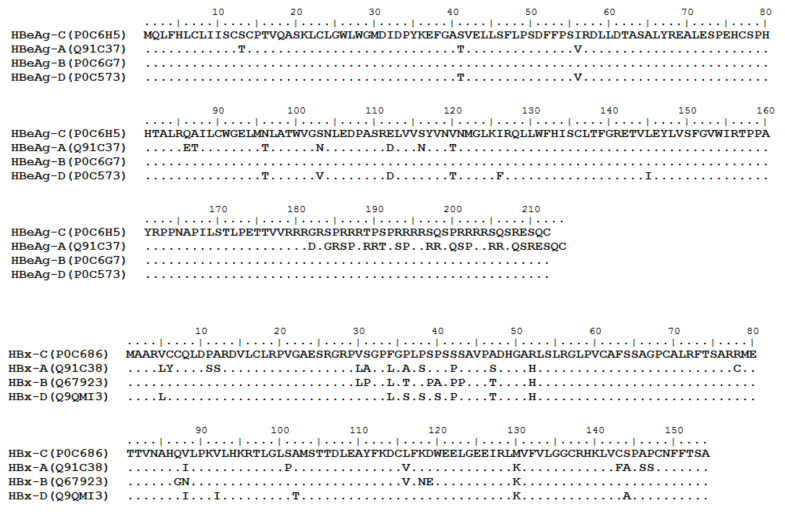

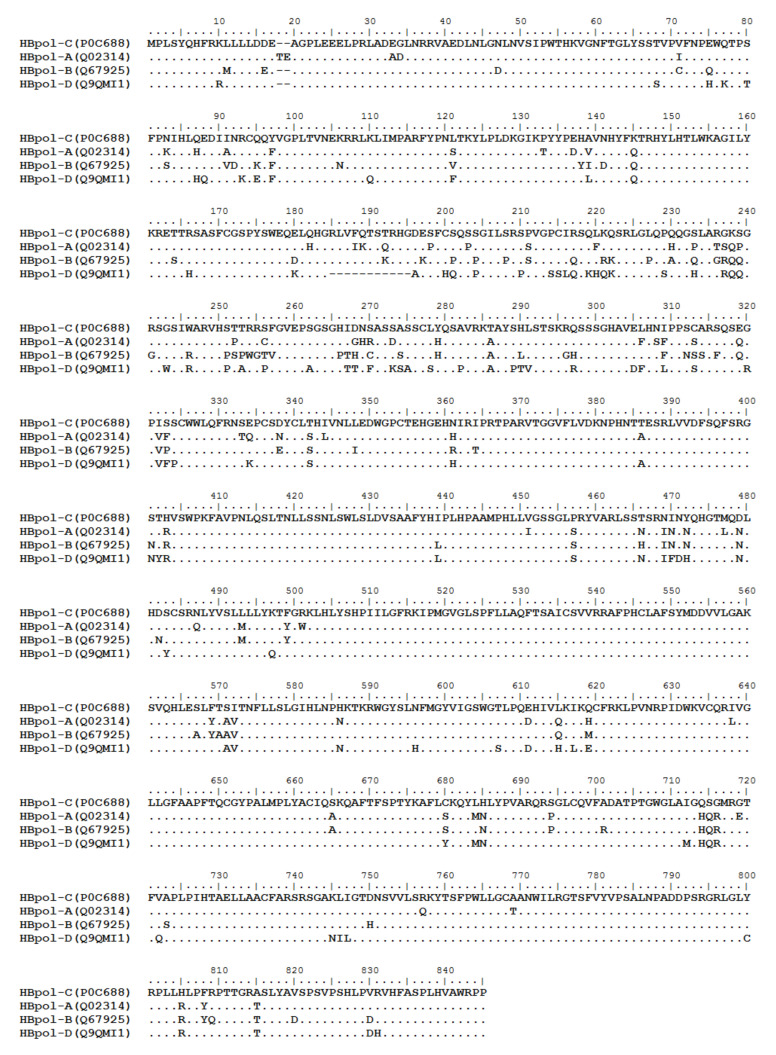
Homologous analysis of HBsAg, HBeAg, HBx and HBpol proteins from HBV C, A, B, and D genotypes. The entire amino acid sequences of each protein from different HBV genotypes were obtained from the UniProt database, aligned and used for in silico prediction of HBV antigen T cell epitopes presented by HLA-A allotypes.

HBV is one of the smallest viruses with a genome length of 3.2 Kb [34]. Its genome contains four open reading frames (ORFs) coding four partially overlapping proteins as displayed in Figure 1: (1) preS/S ORF encodes large (L), middle (M), and small (S) surface antigens (HBsAg). HBsAg is being widely investigated in clinical fields and quantified as a diagnostic marker of HBV infection as it can reflect the level of covalently closed circular DNA (cccDNA) and intrahepatic HBV DNA in chronic infection [35,36]. (2) Pre-core/core ORF encodes hepatitis B e antigen (HBeAg), core antigen (HBcAg) or in combination core-related antigen (HBcrAg). HBeAg has long been advocated as a serum marker for guiding the clinical practice of chronic hepatitis B virus [37,38]. HBcrAg has been demonstrated more recently as a potential surrogate marker of cccDNA [39]. (3) X ORF encodes HBx antigen (HBxAg), which plays an important role in virus genome transcription and is correlated with liver cancer. The expression of HBxAg in HBV-associated HCC patients is significantly higher than other viral proteins [40]. (4) P ORF encodes the viral DNA polymerase (HBpol), which is responsible for the replication of the viral genome and is an effective target for the therapeutic intervention of chronic HBV infection [41]. Human HBV strains occur in nine genotypes A-I, and its major HBV surface antigen (HBsAg) has several immune protective conformational B cell epitopes a, d or y, w1–4 or r [42]. The entire amino acid sequences of each protein from different genotypes were obtained from the UniProt database and aligned in Figure 2.

The process of T cell epitope identification begins with the selection of candidate epitope peptides. The first strategy is using overlapping peptides (OLPs) spanning the entire proteome or selected antigens of interest (peptide scanning). Chen et al. expanded HBV-specific T cells in vitro by co-culturing the overlapping peptide pools spanning the entire sequence of HBV genotypes B and C and the peripheral blood mononuclear cells (PBMCs) from patients with chronic HBV infection, followed by the detection of T cell response in each co-culture using IFN-γ enzyme-linked immunospot (IFN-γ ELISpot) assay, IFN-γ intracellular staining and flow cytometry [43]. However, peptide scanning is a high-cost and laborious method due to a large amount of OLPs spanning overall HBV proteins. For CD8^+^ T cell epitopes, HBsAg, HBeAg, HBx and HBpol contain 131, 68, 49, and 279 OLPs, respectively, when overlapping 6 amino acids in each 9-mer peptide. An alternative strategy is to focus on the in silico predicted T cell epitopes binding to the indicated HLA supertypes as calculated by multiple epitope prediction tools and algorithms. Brinck-Jensen et al. predicted 20 HBV-specific epitopes using combined in silico methods and evaluated for the immunogenicity of these epitopes through exposure to patients’ PBMCs by IFN-γ ELISpot [44]. More recently, a similar in silico approach was also employed to assess all previously verified HBx- and HBpol-derived epitopes and to predict novel HLA-binding peptides for 6 HLA supertypes. Then, a part of reported epitopes were chosen for experimental validation. A total of 13 HLA binders derived from HBx and 33 binders from HBpol were described across HLA subtypes by this strategy [45]. Predicted epitopes are based on the indicated HLA restrictions and limit the number of research objects with diverse HLA subtypes to a reasonable range, yet the inaccuracy of theoretical prediction may omit some real-world epitopes. 

The methodologies to validate the immunogenicity of candidate epitope peptides have been improved remarkably over the last two decades. Different assays are utilized for the detection of peptide-induced T cell response or peptide-specific T cells with individual advantages and disadvantages in terms of practicability, cost, sensitivity, function evaluation. The following approaches are currently widely used, such as cytotoxicity assay, proliferation assay, intracellular cytokine staining (ICS), ELISpot/FluoroSpot, and peptide-MHC multimers staining (tetramers, pentamers, or dextramers). The cytotoxicity assay was initially performed to validate CD8^+^ T cell epitopes by co-culturing patients’ PBMCs with target cells labeled with Chromium-51, after the PBMCs were stimulated by the indicated candidate epitope peptides [46]. Additionally, lymphocyte proliferation assay is mostly applied to CD4^+^ T cell epitopes validation. The PBMCs from HBV-infected or HBV-vaccinated individuals were co-cultured with HBV-derived peptides for several days and ^3^H-thymidine pulses were administered eventually followed by quantifying the incorporated radioactivity [47]. One more common approach currently utilized is ICS or ELISpot/FluoroSpot. Patient’s PBMCs are in vitro or ex vivo stimulated with the candidate epitope peptides and simultaneously cytokine release is blocked followed by ICS and flow cytometry to define whether CD4^+^ T cells or CD8^+^ T cells activation [48]. The ELISpot or FluoroSpot technology enables the detection of single activated cells among one million PBMCs. The accuracy, sensitivity, reproducibility and durability have led to its widespread applications in researches and the broad prospects in the clinical detection of antigen-specific T cells [49,50]. An issue encountered with ELISpot, FluoroSpot, ICS, and related assays is that they may ignore T cells that produce different cytokines or trace cytokines during the window of time of the assay (e.g., Follicular helper CD4^+^ T cells generally produce very low amounts of cytokines). Peptide-MHC tetramer staining has been the gold standard to quantify antigen-specific T cells with high sensitivity and precision, thus is often used to identify T cell epitopes in many researches. However, the preparation of peptide-MHC tetramers or multimers is high-cost, complicated, and time-consumption [51,52]. A pioneering study focused on all possible peptides of the entire HBV genome and 484 unique HLA-A1101-restricted epitopes predicted by NetMHC algorithms were validated using mass cytometry and multiplex peptide-tetramers staining [53]. Many researchers also have established a transgenic mouse model to map HLA-restricted epitopes. Ru et al. developed and immunized HLA-A2/DP4 mice with epitopes derived from HBsAg to identify four new HLA-DP4-restricted epitopes [54]. Besides cellular functional experiments, peptide-HLA molecule binding and stabilization assays were commonly used to identify epitopes. Pan et al. defined 16 HBV epitopes by analyzing the different binding affinities of candidate epitope peptides with HLA-A3303 using RMA-S cells binding and stability assay. More recently, Ferretti et al. used a high-throughput genome-wide screening technology to identify the target cells expressing candidate epitopes productively recognized by T cells (T-Scan) and determined 29 epitopes in SARS-CoV-2 for the six most prevalent HLA types [55]. Chikata et al. employed immunocapture and liquid chromatography mass spectrometry (LC-MS) subsequent to pre-treatment of the target protein to disrupt its three-dimensional structure to characterize HIV-1 epitope peptides on a large scale presented by HLA-C1202 [56]. A variety of epitope assay strategies have been utilized with their own features and potential. 

## 4. Defined T Cell Epitopes in HBV Proteins during the Past 33 Years

Table 1 collected the CD8^+^ T cell epitopes and CD4^+^ T cell epitopes defined from HBV proteome during the past 33 years and displayed their HLA restrictions and the methods used to validate their immunogenicity. Notably, we performed manual management in this review, only the epitopes of 8–14 or 12–25 amino acids in length presented by HLA class I molecules or class II molecules are displayed since they reflect the standard size of the peptide-binding groove of HLA molecules. According to the previous report, if the epitope peptides are too short or long, the experiment tends to represent false positives instead of the result caused by the binding of peptide and HLA molecule [57]. 

**Figure 3:**
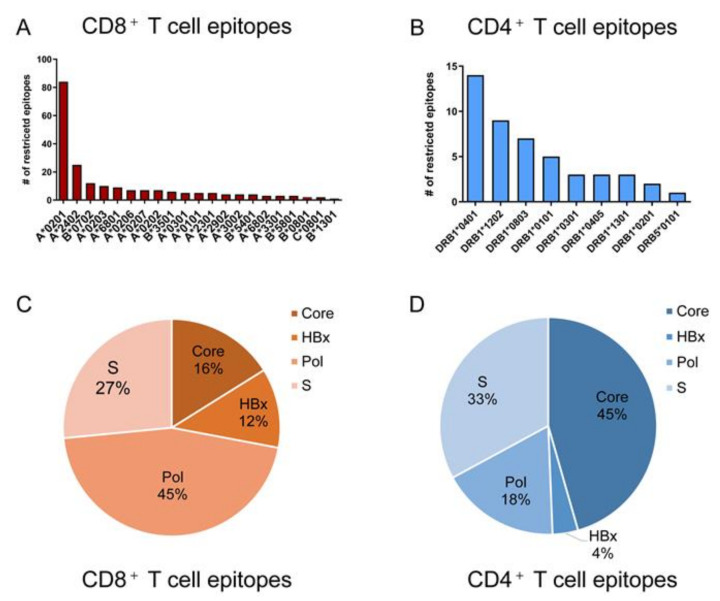
HLA restriction and protein distribution of validated CD4+ T cell epitopes and CD8+ T cell epitopes in HBV proteome. (**A**,**B**) displayed the number of CD8+ T cell epitopes and CD4+ T cell epitopes restricted by each HLA supertype, respectively. (**C**,**D**) showed the fraction of CD8+ T cell epitopes and CD4+ T cell epitopes in each HBV protein, respectively.

Overall, 82 and 19 studies reported the epitopes presented by HLA class I molecules and class II molecules, respectively, and totally contained 284 unique epitopes including 205 CD8^+^ T cell epitopes and 79 CD4^+^ T cell epitopes (Table 1). Of these, 121 (59.0%) CD8^+^ T cell epitopes are restricted by HLA-A0201, A2402 or B0702 (Figure 3A), which are common supertypes in Caucasians and less predominant in Asia and Africa [58,59]. The remainder are restricted mainly by 12 HLA-A, 5 HLA-B and 1 HLA-C supertypes. For the CD4^+^ T cell epitopes, the majority of currently described restrictions apply to 8 DRB1 supertypes (Figure 3B). The cumulative frequency of the HLA-A supertypes described in Figure 3A was highest in Europe (66.6%), followed by Asia (53.1%), Africa (50.7%), and North America (52.3%) while the HLA-B supertypes showed an accumulative frequency of 32.7% in Europe, 20.1% in Asia, 19.2% in Africa and 18.8% in North America. The DRB1 supertypes in Figure 3B displayed little difference in the cumulative gene frequency in Europe (30.5%), Asia (32.2%), Africa (31.1%) and North America (34.1%). (Data from http://www.allelefrequencies.net/, assessed on 11 November 2021). Obviously, the 284 validated T cell epitopes of HBV cannot cover the major populations in an indicated geographic region. More T cell epitopes restricted by more HLA supertypes are urgently needed. Further efforts are required to identify more T cell epitopes restricted to the regional prevalent HLA supertypes, especially for the HLA alleles prevalent in Asian populations with a high HBV incidence [59,60].

In addition, although the validated T cell epitopes have derived from all HBV proteins, the CD8^+^ T cell epitopes mainly distribute in HBpol and HBsAg (72%) (Figure 3C), while the majority of CD4^+^ T cell epitopes concentrate in HBeAg and HBsAg (78%) (Figure 3D). The biased distribution of epitopes in proteome may be caused partially by the different lengths of proteins (HBpol 843aa, HBsAg 400 aa, HBeAg 212 aa, HBx 154 aa) and the pitfalls of screening methods.

As displayed in the sixth column of Table 1, most studies used the in silico prediction strategy to screen the candidate CD8^+^ T cell epitopes (92% of studies) and CD4^+^ T cells epitopes (63% of studies). Relatively, overlapping peptides were more often used in selecting candidate CD4^+^ T cell epitopes (7 of 19 studies; 37%) than CD8^+^ T cell epitopes (7 of 82 studies; 8%), partially due to the lower accuracy and efficacy of predicting HLA class II molecule-binding epitopes as compared with class I molecule-binding epitopes.

**Table 1:** List of CD4^+^ T cell epitopes and CD8^+^ T cell epitopes validated from HBV proteins.

Sequence	Protein	Position	Reference	HLA Restriction	Method to Screen Candidate Epitopes	Method to Validate the Candidate Epitopes
MQLFHLCLI	Core	1–8	[61]	A*0201	Predicted	Binding assay; ELISpot; Cytotoxicity assay; CTL assay
KEFGASVEL(L)	Core	7–15/16	[62]	A*0206, B*4001	Predicted	ELISpot; ICS; Binding assay
EFGASVELL	Core	8–16	[63]	A*0201, A*0207	overlapping	ICS; ELISpot
FLPSDFFPS	Core	18–26	[64]	A*0201	Predicted	ICS; Tetramer staining
FLPSDFFPSV	Core	18–27	[45,65,66,67,68,69,70,71,72,73,74,75,76,77,78,79]	A*02, A*0201, A*0202, A*0203, A*0206, A*6802, A*0301, A*0207	overlapping	Immunization of mice; Cytotoxicity assay; CTL assay; Binding assay; Tetramer staining; ELISA
LPSDFFPSV	Core	19–27	[74,80,81,82,83]	B*3501, B*51, B*5301, B*5401, B*07, B*51, A*0201	overlapping	Binding assay; CTL assay; Cytotoxicity assay; Tetramer staining
FFPSIRDLL	Core	23–31	[84]	A*24	Predicted	Tetramer staining
DLLDTASALY	Core	39–48	[81]	A*0101, A*2902, A*3002	Predicted	Binding assay; Immunization of mice; ELISpot
DFFPSIRDL	Core	51–59	[85]	A*2402	Predicted	ELISpot
LCWGELMNL	Core	60–68	[86]	A*0201	Predicted	Stabilization assay; ELISpot assay
ELMNLATWV	Core	64–72	[87]	A*02	Predicted	Binding assay; ELISpot; Cytotoxicity assay
SYVNMNMGL	Core	87–95	[88]	A*2402	Predicted	Binding assay; CTL assay
SYVNTNMGL	Core	87–95	[89]	A*02	Predicted	Tetramer staining
YVNVNMGLK	Core	88–96	[63]	A*1101	overlapping	ICS; ELISpot
MGLKFRQL	Core	93–100	[90]	A*0201	Predicted	Immunization of mice; FACS
LLWFHISCL	Core	101–108	[43]	A*0201	Predicted	Proliferation assay; ICS; Cytotoxicity assay
LWFHISCLTF	Core	101–110	[85]	A*2402, A*2301	Predicted	ELISpot
HISCLTFGR	Core	104–112	[91,92]	A*33	Predicted	Cytotoxicity assay; ICS; Tetramer staining
CLTFGRETV	Core	107–115	[93]	A*02	Predicted	Tetramer staining
EYLVSFGVW	Core	117–125	[81,84,88]	A*2402, A*2407, A*2301	Predicted	Stabilization assay; CTL assay; Cytotoxicity assay; Tetramer staining; Binding assay; Immunization of mice; ELISpot
YLVSFGVWI	Core	118–126	[43]	A*0201	Predicted	Proliferation assay; ICS; Cytotoxicity assay
LVSFGVWIR	Core	119–127	[91]	A*33	Predicted	Stabilization assay; ELISpot; Cytotoxicity assay; Immunization of mice
GLKILQLL	Core	123–130	[82]	B*08	overlapping	ICS; Tetramer staining
AYRPPNAPI	Core	131–139	[94]	A*0201	Predicted	ELISpot; Cytotoxicity assay
LTFGRETVLEN	Core	137–147	[95]	A*0101, A*02, A*2902, A*3002	Predicted	ELISpot
ILSTLPETTV	Core	139–148	[75]	A*02	Predicted	CTL assay
STLPETTVVR	Core	141–150	[74,96]	A*11, A*6801, A*02	overlapping	Binding assay; CTL assay; Cytotoxicity assay; ELISpot
STLPETTVVRR	Core	141–151	[17,76,81,92,97]	A*31, A*68, A*02, A*0201, A*6801, A*03, A*11, A*3101, A*0201	overlapping	Cytotoxicity assay; Immunization of mice; CTL assay; Binding assay; ELISpot
TLPETTVVRR	Core	142–151	[63]	A*1101	overlapping	ICS; ELISpot
GVWIRTPPA	Core	152–160	[98]	A*0201	Predicted	ELISpot
STLPETAVVRR	Core	170–180	[9]	A*1101	Predicted	Proliferation assay; Tetramer staining
RTQSPRRR	Core	196–203	[9]	A*1101	Predicted	Proliferation assay; Tetramer staining
RTQSPRRRR	Core	196–204	[9]	A*1101	Predicted	Proliferation assay; Tetramer staining
RSQSPRRRRSK	Core	196–206	[9]	A*1101	Predicted	Proliferation assay; Tetramer staining
RLCCQLDPA	HBx	4–12	[99]	A*0201	Predicted	Binding assay; ELISpot; Cytotoxicity assay
AYFKDCVFKDW	HBx	6–16	[45]	A*2402	Predicted	ELISA
QLDPARDVL	HBx	8–16	[45,65,73,99,100,101]	A*0201	Predicted	ELISpot
VLCLRPVGA	HBx	15–23	[45,99,102]	A*0201	Predicted	ELISpot
RGRPVSGPF	HBx	26–34	[85]	A*2402	Predicted	ELISpot
PVSGPFGPL	HBx	29–37	[100]	A*0201	Predicted	Immunization of mice; CTL assay; Cytotoxicity assay
AVPADHGAHL	HBx	44–53	[100]	A*0201	Predicted	Immunization of mice; CTL assay; Cytotoxicity assay
HLSLRGLPV	HBx	52–60	[65,99,100,101,102,103]	A*0201, A*02	Predicted	Cytotoxicity assay; Immunization of mice; CTL assay; Binding assay; ELISpot
LPVCAFSSA	HBx	58–66	[45]	B*0702	Predicted	ELISA
AFSSAGPCALRF	HBx	62–73	[45]	A*2402	Predicted	ELISA
ALRFTSARR	HBx	70–78	[45]	A*0301	Predicted	ELISA
ALRFTSARRM	HBx	70–79	[100]	A*0201	Predicted	Immunization of mice; CTL assay; Cytotoxicity assay
NAHQILPKV	HBx	84–92	[99]	A*0201	Predicted	Binding assay; ELISpot; Cytotoxicity assay
(K)VLHKRTLGL	HBx	91/92–100	[65,100,102]	A*0201	Predicted	Cytotoxicity assay; Binding assay; ELISpot; Tetramer staining
VLHKRTLGL	HBx	92–100	[99,101,104]	A*0201, A*02	Predicted	Binding assay; ELISpot; Cytotoxicity assay; Proliferation assay; ELISpot; ICS
TLGLAAMST	HBx	97–105	[100]	A*0201	Predicted	Binding assay; ELISpot; Cytotoxicity assay
GLSAMSTTDL	HBx	99–108	[99,100,104]	A*0201, A*02	Predicted	Binding assay; ELISpot; Cytotoxicity assay
AMSTTDLEA	HBx	102–110	[99]	A*0201	Predicted	Binding assay; ELISpot; Cytotoxicity assay
STTDLEAYFK	HBx	104–113	[45]	A*1101	Predicted	ELISA
DLEAYFKDCL	HBx	107–116	[100]	A*0201	Predicted	Immunization of mice; CTL assay; Cytotoxicity assay
CLFKDWEEL	HBx	115–123	[99,100,102]	A*0201	Predicted	Immunization of mice; CTL assay; Cytotoxicity assay; Binding assay; ELISpot
ELGEEIRLKV	HBx	122–131	[100]	A*0201	Predicted	Immunization of mice; CTL assay; Cytotoxicity assay
EIRLKVFVL	HBx	126–134	[100]	A*0201	Predicted	Immunization of mice; CTL assay; Cytotoxicity assay
VLGGCRHKL	HBx	133–141	[99,101]	A*0201, A*02	Predicted	Binding assay; ELISpot; Cytotoxicity assay; ELISpot
VLGGCRHKL(V)	HBx	133–141/142	[98]	A*0201	Predicted	Immunization of mice; CTL assay; Cytotoxicity assay
LLDDEAGPL	Pol	13–21	[105,106]	A*0201	Predicted	Binding assay; Immunization of mice; CTL assay; Cytotoxicity assay
PLEEELPRL	Pol	20–28	[105,106]	A*0201	Predicted	Binding assay; Immunization of mice; CTL assay; Cytotoxicity assay
DLNLGNLN	Pol	40–48	[106]	A*0201	Predicted	Binding assay; Immunization of mice; CTL assay; Cytotoxicity assay
NLGNLNVSI	Pol	42–50	[106]	A*0201	Predicted	Binding assay; Immunization of mice; CTL assay; Cytotoxicity assay
NVSIPWTHK	Pol	47–55	[9,74,81]	A*03, A*11, A*6801, A*0301, A*1101	Predicted	Stabilization assay; ELISpot; Cytotoxicity assay; Immunization of mice; Proliferation assay; Tetramer staining; Binding assay
KVGNFTGLY	Pol	55–63	[45,74]	A*0301, A*03, A*11	Predicted	Binding assay; CTL assay; Cytotoxicity assay; ELISA
GLYSSTVPV	Pol	61–69	[73,105,106]	A*0201	Predicted	Binding assay; Immunization of mice; CTL assay; Cytotoxicity assay; Tetramer staining
LYSSTVPVF	Pol	62–70	[79]	A*24	Predicted	ELISpot
STVPCFNPK	Pol	65–73	[9]	A*1101	Predicted	Proliferation assay; Tetramer staining
TVPCFNPK	Pol	66–73	[9]	A*1101	Predicted	Proliferation assay; Tetramer staining
PSFPHIHLK	Pol	77–85	[9]	A*1101	Predicted	Proliferation assay; Tetramer staining
QYVGPLTVN	Pol	94–102	[85]	A*2402	Predicted	ELISpot
YLHTLWKAGI	Pol	147–156	[65]	A*02	Predicted	ELISpot assay; Tetramer staining
(H)TLWKAGILYK	Pol	149/150–159	[81]	A*03	Predicted	Binding assay; Immunization of mice; ELISpot
HTLWKAGILYK	Pol	149–159	[74,76,98]	A*03, A*11, A*3101, A*3301, A*6801, A*02, A*11	Predicted	Immunization of mice; Cytotoxicity assay; Binding assay; CTL assay
TLWKAGILY(K)	Pol	150–158/159	[74]	A*03, A*11	Predicted	Binding assay; CTL assay; Cytotoxicity assay
RSASFCGSPY	Pol	164–173	[45]	A*1101	Predicted	ELISA
ASFCGSPYSW	Pol	166–175	[45,62,63]	A*2402, B*5801	overlapping	ELISA; ELISpot; ICS
SFCGSPYSW	Pol	167–175	[45]	A*2402	Predicted	ELISA
ASFCGSPY	Pol	166–173	[81,95,107]	A*0101, A*2902, A*3002	overlapping	Binding assay; Immunization of mice; ELISpot; Tetramer staining
SPYSWEQEL	Pol	171–179	[17]	A*0201, B*3501	Predicted	Tetramer staining
QSSGILSR	Pol	200–207	[9]	A*1101	Predicted	Proliferation assay; Tetramer staining
GILPRSSVGPR	Pol	205–215	[9]	A*1101	Predicted	Proliferation assay; Tetramer staining
CLHQSAVRK	Pol	274–282	[45]	A*0301, A*1101	Predicted	ELISA
KTAYSHLSTSK	Pol	283–293	[9]	A*1101	Predicted	Proliferation assay; Tetramer staining
SSARSQSER	Pol	310–318	[9]	A*1101	Predicted	Proliferation assay; Tetramer staining
CLSLIVNLL	Pol	338–346	[65]	A*02	Predicted	ELISpot assay; Tetramer staining
TPARVTGGV	Pol	354–362	[45]	B*0702	Predicted	ELISA
TPARVTGGVF	Pol	354–363	[45]	B*0702	Predicted	ELISA
RVTGGVFLV	Pol	357–365	[45]	A*0201	Predicted	ELISA
VTGGVFLVDK	Pol	358–367	[45]	A*1101, A*03	Predicted	ELISA
RIPRTPSRV	Pol	361–369	[65]	A*02	Predicted	ELISpot assay; Tetramer staining
TPARVTGGVF	Pol	365–374	[74,76,108]	B*0702, B*3501, A*03, B*07, A*02, B*51	Predicted	Immunization of mice; Cytotoxicity assay; Binding assay; CTL assay
RVTGGVFLVDK	Pol	368–378	[74]	A*11	Predicted	Binding assay; CTL assay; Cytotoxicity assay
VTGGVFLVDK	Pol	369–378	[74]	A*03, A*11	Predicted	Binding assay; CTL assay; Cytotoxicity assay
FLVDKNPHNT	Pol	374–383	[62]	A*0203	Predicted	ELISpot; ICS; Binding assay
LVVDFLHQFSR	Pol	377–386	[9]	A*1101, A*3301, A*6801	Predicted	Proliferation assay; Tetramer staining; Binding assay; Immunization of mice; ELISpot; CTL assay; Cytotoxicity assay
SRLVVDFSQF	Pol	386–395	[63]	B*1301	overlapping	ICS; ELISpot
VVDFSQFSR	Pol	389–397	[74,91]	A*11, A*6801, A*33	Predicted	Stabilization assay; ELISpot; Cytotoxicity assay; Binding assay; Immunization of mice; CTL assay
SWPKFAVPNL	Pol	392–401	[45]	A*2402	Predicted	ELISA
WPKFAVPNL	Pol	393–401	[45]	B*0702	Predicted	ELISA
FAVPNLQSL	Pol	396–404	[45]	A*0201	Predicted	ELISA
NLQSLTNLL	Pol	411–419	[105,106]	A*0201	Predicted	Cytotoxicity assay; Immunization of mice; Binding assay; CTL assay
LLSSNLSWL	Pol	418–426	[65,105,106]	A*0201	Predicted	Cytotoxicity assay; Immunization of mice; Binding assay; CTL assay; ELISpot; Tetramer staining
NLSWLSLDV	Pol	422–430	[101,105,106]	A*0201, A*02	Predicted	Cytotoxicity assay; Immunization of mice; Binding assay; CTL assay; ELISpot
LSLDVSAAFY	Pol	426–435	[81]	A*0101, A*2902, A*3002	Predicted	Binding assay; Immunization of mice; ELISpot
HPAAMPHLL	Pol	440–448	[74]	B*0702	Predicted	Binding assay; CTL assay; Cytotoxicity assay
HLLVGSSGL	Pol	446–454	[105,106]	A*0201	Predicted	Cytotoxicity assay; Immunization of mice; Binding assay; CTL assay
GLPRYVARL	Pol	453–461	[65,71,73,74,81,92,93,100,101,106,109,110,111]	A*0201, A*0202, A*0203, A*02, A*0207	Predicted	Cytotoxicity assay; Immunization of mice; Binding assay; CTL assay; ELISpot; Tetramer staining
RIINNQHR	Pol	466–473	[9]	A*1101	Predicted	Proliferation assay; Tetramer staining
RNLYVSLLL	Pol	484–492	[85]	A*2402	Predicted	ELISpot
NLYVSLLLL	Pol	485–493	[65,106]	A*0201, A*02	Predicted	Cytotoxicity assay; Immunization of mice; Binding assay; CTL assay; ELISpot; Tetramer staining
KLHLYSHPI	Pol	500–508	[45,62,93,101,106]	A*0201, A*02, A*0203, B*0801	Predicted	Cytotoxicity assay; Immunization of mice; Binding assay; CTL assay; ELISpot; Tetramer staining; ELISA
HLYSHPIIL	Pol	502–510	[65,105,112,113,114]	A*0201, A*02, A*0203	overlapping	Cytotoxicity assay; Immunization of mice; Binding assay; ELISpot; Tetramer staining
IPMGVGLSP	Pol	504–512	[45]	B*0702	Predicted	ELISA
ILGFRKIPM	Pol	509–517	[45]	B*0801	Predicted	ELISA
FLLAQFTSAI	Pol	524–533	[65,101]	A*0201, A*02	Predicted	ELISpot; Tetramer staining
LLAQFTSAI	Pol	525–533	[65,101,106]	A*0201, A*02	Predicted	Cytotoxicity assay; Immunization of mice; Binding assay; ELISpot; Tetramer staining
SAICSVVRR	Pol	531–539	[74]	A*11, A*3301, A*6801	Predicted	Binding assay; CTL assay; Cytotoxicity assay
SVVRRAFPH	Pol	535–542	[9]	A*1101	Predicted	Proliferation assay; Tetramer staining
FFPHCLAFSYM	Pol	539–550	[81]	B*07	Predicted	Binding assay; Immunization of mice; ELISpot
FPHCLAFSYM	Pol	540–550	[74]	B*0702, B*3501, B*51, B*5301, B*5401	Predicted	Binding assay; CTL assay; Cytotoxicity assay
YMDDVVLG	Pol	549–556	[81]	A*0201, A*0202, A*0203, A*0206, A*6802	Predicted	Binding assay; Immunization of mice; ELISpot
YMDDVVLGA	Pol	549–557	[45,71,72,99,101,114,115,116]	A*0201, A*02, A*0101	overlapping	Cytotoxicity assay; Immunization of mice; Binding assay; ELISpot; CTL assay; ELISA
YMDDVVLGAK	Pol	549–558	[74]	A*03	Predicted	Binding assay; CTL assay; Cytotoxicity assay
FLLSLGIHL	Pol	573–581	[71,73,74,81,93,106,108,110,116,117,118,119,120]	A*02, A*0201, A*0206, A*0202	Predicted	Cytotoxicity assay; Immunization of mice; Binding assay; ELISpot; CTL assay; Tetramer staining
SLNFMGYVI	Pol	592–600	[106]	A*0201	Predicted	Binding assay; Immunization of mice; CTL assay; Cytotoxicity assay
PVNRPIDWK	Pol	612–620	[9]	A*1101	Predicted	Proliferation assay; Tetramer staining
PVNRPIDWK	Pol	623–631	[74]	A*03, A*11	Predicted	Binding assay; CTL assay; Cytotoxicity assay
CGYPALMPLY	Pol	638–647	[45]	A*2402	Predicted	ELISA
GYPALMPLY	Pol	639–647	[45]	A*2402	Predicted	ELISA
YPALMPLYA	Pol	651–659	[74]	B*0702, B*3501, B*51, B*5401	Predicted	Binding assay; CTL assay; Cytotoxicity assay
YPALMPLSA	Pol	651–659	[62]	B*5401	Predicted	ELISpot; ICS; Binding assay
ALMPLYACI	Pol	653–661	[71,74,93,106]	A*0201, A*0202, A*0203, A*0204, A*0206, A*02	Predicted	Cytotoxicity assay; Immunization of mice; Binding assay; ELISpot; CTL assay; Tetramer staining
QAFTFSPTYK	Pol	665–674	[74,113]	A*03, A*11, A*6801	Predicted	Cytotoxicity assay; Binding assay; CTL assay
VFADATPTGW	Pol	686–695	[45]	A*2402	Predicted	ELISA
GLCQVFADA	Pol	692–700	[45]	A*0201	Predicted	ELISA
LPIHTAELL	Pol	712–720	[45]	B*0702	Predicted	ELISA
PLPIHTAEL	Pol	722–730	[106]	A*0201	Predicted	Binding assay; Immunization of mice; CTL assay; Cytotoxicity assay
IIGTDNSVV	Pol	744–752	[65]	A*0201	Predicted	ELISpot assay; Tetramer staining
RKYTSFPWLL	Pol	744–753	[45]	A*2402	Predicted	ELISA
KYTSFPWLLG	Pol	745–754	[45]	A*2402	Predicted	ELISA
GTDNSVVLSR	Pol	746–755	[74]	A*11	Predicted	Binding assay; CTL assay; Cytotoxicity assay
KYTSFPWLL	Pol	756–764	[63,81,84,88,93]	A*24, A*2301, A*2402	overlapping	Cytotoxicity assay; Immunization of mice; Binding assay; ELISpot; CTL assay; Tetramer staining; ICS; ELISA
LLGCAANWI	Pol	763–771	[65,106]	A*0201	Predicted	Cytotoxicity assay; Immunization of mice; Binding assay; ELISpot; CTL assay; Tetramer staining
WILRGTSFV	Pol	770–778	[65,105]	A*0201, A*02	Predicted	Immunization of mice; Binding assay; ELISpot; Tetramer staining
ILRGTSFVYV	Pol	771–780	[65,71]	A*0201, A*02	Predicted	Cytotoxicity assay; ELISpot; Tetramer staining
DPSRGRLGL	Pol	789–797	[74]	B*0702	Predicted	Binding assay; CTL assay; Cytotoxicity assay
RLGLSRPLL	Pol	794–802	[106]	A*0201	Predicted	Binding assay; Immunization of mice; CTL assay; Cytotoxicity assay
GLSRPLLRL	Pol	796–804	[65]	A*02	Predicted	ELISpot assay; Tetramer staining
LVYRPTTGR	Pol	804–812	[9]	A*1101	Predicted	Proliferation assay; Tetramer staining
SLYADSPSV	Pol	814–822	[65,71,73,90,93,106,114,116]	A*0201, A*02	Predicted	Cytotoxicity assay; Immunization of mice; Binding assay; ELISpot; CTL assay; Tetramer staining; FACS
FLLTRILTI	S	20–28	[66,67,68,77,100,121]	A*0201	Predicted	ICS; Tetramer staining; Cytotoxicity assay; Degranulation assay
PLGFFPDH	S	21–28	[122]	A*11	Predicted	ELISpot
NLLGWSPQA	S	73–81	[63]	A*0201, A*0207	overlapping	ICS; ELISpot
LTTVPAASLLA	S	85–95	[95]	A*02	Predicted	ELISpot
TTSTGPCK	S	115–122	[9]	A*1101	Predicted	Proliferation assay; Tetramer staining
LLDPRVRGL	S	131–139	[75]	A*02	Predicted	CTL assay
AILSKTGDPV	S	160–169	[116]	A*02	Predicted	Tetramer staining
FLGPLLVLQA	S	182–190	[62,63,107]	C*0801	overlapping	Cytotoxicity assay; Binding assay; ELISpot; Tetramer staining;
VLQAGFFL	S	188–195	[62]	C*0801	Predicted	ELISpot; ICS; Binding assay
VLQAGFFLL	S	188–196	[65,73,101,116,123]	A*0201, A*02	Predicted	Cytotoxicity assay; Immunization of mice; Binding assay; ELISpot; CTL assay; Tetramer staining
SWWTSLNFL	S	192–200	[85]	A*2402	Predicted	ELISpot
FLLTRILTI	S	194–202	[54,74,76,81,90,93,94,101,108,111,114,116,119,120,123,124,125,126,127,128]	A*0201, A*0202, A*0203, A*0206, A*02	overlapping	Cytotoxicity assay; Immunization of mice; Binding assay; ELISpot; CTL assay; Tetramer staining; ICS; FACS
IPQSLDSWWTSL	S	202–213	[129,130]	A*0201, A*02	Predicted	Cytotoxicity assay; Immunization of mice; Binding assay; ELISpot
SILSPFLPLL	S	207–216	[131]	A*0201	Predicted	Binding assay; ELISpot
NILSPFMPLL	S	207–216	[131]	A*0201	Predicted	Binding assay; ELISpot
ILSPFMPLL	S	208–216	[131]	A*0201	Predicted	Binding assay; ELISpot
TLSPFLPLL	S	208–216	[131]	A*0201	Predicted	Binding assay; ELISpot
SWWTSLNFL	S	208–216	[84]	A*24	Predicted	Tetramer staining
FLGGTPVCL	S	215–223	[95,116,123,125]	A*0201A*02, A*24	Predicted	Cytotoxicity assay; Immunization of mice; Binding assay; ELISpot; CTL assay; Tetramer staining
SWLSLLVPF	S	226–234	[85]	A*2402	Predicted	ELISpot
RWMCLRRFII	S	236–245	[85]	A*2402	Predicted	ELISpot
CPGYRWMCL	S	243–251	[108]	B*07	Predicted	Cytotoxicity assay
GYRWMCLRR	S	245–253	[91]	A*33	Predicted	Stabilization assay; ELISpot; Cytotoxicity assay; Immunization of mice
RWMCLRRFII	S	247–256	[81]	A*2301, A*2402	Predicted	Binding assay; Immunization of mice; ELISpot
ILLLCLIFL	S	260–268	[73,125]	A*0201	Predicted	Cytotoxicity assay; Immunization of mice
LLLCLIFLL	S	261–268	[72]	A*02	Predicted	Cytotoxicity assay
LLCLIFLLV	S	262–269	[65,115,123]	A*0201, A*02	Predicted	Stabilization assay; ELISpot; Cytotoxicity assay; Tetramer staining; Immunization of mice
LCLIFLLVL	S	263–271	[85]	A*2402	Predicted	ELISpot
(L)VLLDYQGML	S	269/70–278	[75]	A*0201	Predicted	CTL assay
LLDYQGMLP	S	271–279	[123]	A*0201	Predicted	Immunization of transgenic mice; Cytotoxicity assay; ELISpot; Binding assay
LLDYQGMLPV	S	271–280	[72,101,116,125]	A*02	Predicted	ELISpot; Cytotoxicity assay; Binding assay; Tetramer staining
TSMFPSCCCTK	S	305–315	[9]	A*1101	Predicted	Proliferation assay; Tetramer staining
IPIPSSWAF	S	324–332	[74,76,81,108]	B*0702, B*3501, B*51, B*5301, A*03, B*07, A*02, B*5101	Predicted	ELISpot; Cytotoxicity assay; Immunization of mice; Binding assay; CTL assay
YLWEWASVR	S	335–343	[91]	A*33	Predicted	Stabilization assay; ELISpot; Cytotoxicity assay; Immunization of mice
RFSWLSLLVPF	S	343–353	[81]	A*2301, A*2402	Predicted	Binding assay; Immunization of mice; ELISpot
SWLSLLVPF	S	345–353	[84]	A*24	Predicted	Tetramer staining
WLSLLVPFV	S	346–354	[71,72,73,74,75,76,99,105,108,117,118,120,123,132,133]	A*02, A*0201, A*0202, A*0203, A*0206, A*0207, A*04, A*6802	Predicted	ELISpot; Cytotoxicity assay; Immunization of mice; Binding assay; Tetramer staining
LLVPFVQWFV	S	349–358	[93,101,111]	A*02	Predicted	ICS; Degranulation assay; ELISpot; Tetramer staining
VGLSPTVWL	S	358–366	[85]	A*2402	Predicted	ELISpot
GLSPTVWLS	S	359–367	[123]	A*0201	Predicted	Immunization of transgenic mice; Cytotoxicity assay; ELISpot; Binding assay
GLSPTVWLSV	S	359–368	[72,73,90,93,105,111,114,116,124,125,128,130,134]	A*02, A*0201, A*0203, A*0207	overlapping	Immunization of mice; FACS; CTL assay; ELISpot; Tetramer staining; Degranulation assay
VWLSVIWM	S	364–371	[90]	A*0201	Predicted	Immunization of mice; FACS
(L)SVIWMMWYW	S	366/367–375	[62]	B*5801	Predicted	ELISpot; ICS; Binding assay
SVIWMMWYW	S	367–375	[63,107]	B*5801	overlapping	Tetramer staining; ICS; ELISpot
SIVSPFIPLL	S	370–379	[131]	A*0201	Predicted	Binding assay; ELISpot
ILSPFLPLL	S	371–379	[131]	A*0201	Predicted	Binding assay; ELISpot
MMWYWGPSLY	S	371–380	[74]	A*03	Predicted	Binding assay; CTL assay; Cytotoxicity assay
NILSPFLPLL	S	381–390	[131]	A*0201	Predicted	Binding assay; ELISpot
SILSPFLPLL	S	381–390	[77]	A*0201	Predicted	ICS; Tetramer staining;
SIVSPFIPLL	S	381–390	[72,73,116,123]	A*02, A*0201	Predicted	Immunization of mice; FACS; CTL assay; ELISpot; Tetramer staining
ILSPFLPLL	S	382–390	[75,90]	A*0201	Predicted	Immunization of mice; FACS; CTL assay
IVSPFIPLL	S	382–390	[134]	A*0201	Predicted	ELISA; Cytotoxicity assay
ILRSFIPLL	S	382–390	[95]	A*02, A*24	Predicted	ELISpot
LLPIFFCLWV	S	389–398	[101]	A*02	Predicted	ELISpot
DIDPYKEFGATVELL	Core	2–16	[135]	DRB1*0401	overlapping	Proliferation assay; ICS
IDPYKEFGATVELLS	Core	3–17	[135]	DRB1*0401	overlapping	Proliferation assay; ICS
DPYKEFGATVELLSF	Core	4–18	[135]	DRB1*0401	overlapping	Proliferation assay; ICS
PYKEFGATVELLSFL	Core	5–19	[135]	DRB1*0401	overlapping	Proliferation assay; ICS
YKEFGATVELLSFLP	Core	6–20	[135,136]	DRB1*0401, DRB1*1202	overlapping	ICS; Proliferation assay
KEFGATVELLSFLPS	Core	7–21	[135]	DRB1*0401	overlapping	Proliferation assay; ICS
EFGATVELLSFLPSD	Core	8–22	[135]	DRB1*0401	overlapping	Proliferation assay; ICS
FGATVELLSFLPSDF	Core	9–23	[135]	DRB1*0401	overlapping	Proliferation assay; ICS
GATVELLSFLPSDFF	Core	10–24	[135]	DRB1*0401	overlapping	Proliferation assay; ICS
TVELLSFLPSDFFPS	Core	12–26	[135]	DRB1*0401	overlapping	Proliferation assay; ICS
VELLSFLPSDFFPSV	Core	13–27	[135]	DRB1*0401	overlapping	Proliferation assay; ICS
LLSFLPSDFFPSVRD	Core	15–29	[135]	DRB1*0401	overlapping	Proliferation assay; ICS
LSFLPSDFFPSVRDL	Core	16–30	[135]	DRB1*0401	overlapping	Proliferation assay; ICS
FLPSDFFPSVRD	Core	18–29	[137]	DPw4, DRB1*07	Predicted	Cytotoxicity assay
RDLLDTASALYREALESPEH	Core	28–47	[138]	DRB1*07, DPw4	overlapping	Proliferation assay
ALYREALESPEHCSP	Core	36–50	[136]	DRB1*1202	overlapping	ICS
ALESPEHCSPHHTALRQAIL	Core	41–60	[139]	DRB1*13	overlapping	Proliferation assay
EHCSPHHTALRQAIL	Core	46–60	[136]	DRB1*0803	overlapping	ICS
PHHTALRQAILCWGELMTLA	Core	50–69	[81]	DRB1*07, DRB1*09, DRB1*11	Predicted	Binding assay; Immunization of mice; ELISpot
HHTALRQAILCWGEL	Core	51–65	[136]	DRB1*1202	overlapping	ICS
RQAILCWGELMNLAT	Core	56–70	[136]	DRB1*0803, DRB1*1202	overlapping	ICS
LCWGELMTLATWVGVN	Core	60–76	[140]	DRB1*0101	Predicted	Proliferation assay; ICS; Tetramer staining
MNLATWVGSNLEDPA	Core	66–80	[136]	DRB1*0803	overlapping	ICS
LEDPASRELVVSYVN	Core	76–90	[136]	DRB1*1202	overlapping	ICS
SRELVVSYVNVNMGL	Core	81–95	[136]	DRB1*0803	overlapping	ICS
LEYLVSFGVWIRTPP	Core	116–130	[136]	DRB1*1202	overlapping	ICS
EYLVSFGVWIRTPPA	Core	117–131	[138]	DRW52, DRB1*06	overlapping	Proliferation assay
VSFGVWIRTPPAYRPPNAPI	Core	120–139	[81,138]	DRB1*01, DRB1*07, DRB1*11, DRB1*12, DRB1*13	overlapping	Binding assay; Immunization of mice; ELISpot; Proliferation assay
NAPILSTLPETTVVR	Core	136–150	[136]	DRB1*0803	overlapping	ICS
STLPETTVVRRRGRS	Core	141–155	[136]	DRB1*1202	overlapping	ICS
STLPETTVVRRRGRSPRRRT	Core	141–160	[141]	DRB1*13	Predicted	Proliferation assay; Cytotoxicity assay; ICS
PRRRTPSPRRRRSQS	Core	156–170	[136]	DRB1*0803	overlapping	ICS
PPAYRPPNAPILSTL	Core	158–172	[135]	DRB1*0101	overlapping	Proliferation assay; ICS
PAYRPPNAPIL	Core	159–169	[142]	DR52, DRw3	overlapping	Proliferation assay; Cytotoxicity assay
PSPRRRRSQSPRRRR	Core	161–175	[136]	DRB1*0803	overlapping	ICS
RRSQSPRRRRSQSRE	Core	166–180	[136]	DRB1*1202	overlapping	ICS
YFKDCLFKDWEELGE	HBx	111–125	[143]	DRB1*1301	overlapping	ELISpot; Binding assay; ICS
EIRLKVFVLGGCRHK	HBx	126–140	[143]	DRB1*0101, DRB1*0401, DRB1*1301, DRB5*0101	overlapping	ELISpot; Binding assay; ICS
VFVLGGCRHKLVCAP	HBx	131–145	[143]	DRB1*1301	overlapping	ELISpot; Binding assay; ICS
VGPLTVNEKRRLKLI	Pol	96–111	[113]	DRB1*0301	Predicted	ELISpot; Cytotoxicity assay
RHYLHTLWKAGILYK	Pol	145–160	[113]	DRB1*0301, DRB1*07, DRB1*08, DRB1*09, DRB1*11, DRB1*12, DRB1*15	Predicted	ELISpot; Cytotoxicity assay
ESRLVVDFSQFSRGN	Pol	385–400	[113]	DRB1*03, DRB1*04	Predicted	ELISpot; Cytotoxicity assay
LQSLTNLLSSNLSWL	Pol	412–427	[113]	DRB1*01, DRB1*04, DRB1*07, DRB1*11, DRB1*12, DRB1*13, DRB1*15	Predicted	ELISpot; Cytotoxicity assay
SSNLSWLSLDVSAAF	Pol	420–435	[113]	DRB1*01, DRB1*03, DRB1*04, DRB1*13	Predicted	ELISpot; Cytotoxicity assay
LHLYSHPIILGFRKI	Pol	501–516	[113]	DRB1*01, DRB1*04, DRB1*11	Predicted	ELISpot; Cytotoxicity assay
PFLLAQFTSAICSVV	Pol	525–538	[81]	DRB1*01, DRB1*04, DRB1*07, DRB1*08, DRB1*09, DRB1*11, DRB1*15, DRB5*01	Predicted	Binding assay; Immunization of mice; ELISpot
KQCFRKLPVNRPIDW	Pol	618–633	[81,113]	DRB1*01, DRB1*04, DRB1*07, DRB1*13	Predicted	Binding assay; Immunization of mice; ELISpot; Cytotoxicity assay
LCQVFADATPTGWGL	Pol	649–664	[81]	DRB1*03, DRB1*04, DRB1*07	Predicted	Binding assay; Immunization of mice; ELISpot
KQAFTFSPTYKAFLC	Pol	664–679	[113]	DRB1*01, DRB1*04, DRB1*07, DRB1*08, DRB1*09, DRB1*11, DRB1*13, DRB1*15	Predicted	ELISpot; Cytotoxicity assay
AANWILRGTSFVYVP	Pol	676–691	[81]	DRB1*07, DRB1*08, DRB1*09, DRB1*12, DRB1*13, DRB1*15	Predicted	Binding assay; Immunization of mice; ELISpot
LCQVFADATPTGWGL	Pol	694–709	[113]	DRB1*03, DRB1*04	Predicted	ELISpot; Cytotoxicity assay
AANWILRGTSFVYVP	Pol	767–782	[113]	DRB1*01, DRB1*07, DRB1*08, DRB1*09, DRB1*13, DRB1*15	Predicted	ELISpot; Cytotoxicity assay
GTSFVYVPSALNPAD	Pol	774–789	[81]	DRB1*01, DRB1*04, DRB1*07, DRB1*08, DRB1*09, DRB1*11, DRB1*15, DRB5*01	Predicted	Binding assay; Immunization of mice; ELISpot
AGFFLLTRILTIPQS	S	17–31	[144]	DRB1*07, DRB1*08, DRB1*11, DRB1*13	Predicted	ELISpot; Proliferation assay
GFFPDHQLDPAF	S	23–33	[145]	DRB1*0405	Predicted	Binding assay; FASC
TSLNFLGGSPVCLGQ	S	37–51	[144]	DRB1*01	Predicted	ELISpot; Proliferation assay
GAFGPGFTPPHG	S	61–72	[145]	DRB1*0405	Predicted	Binding assay; FASC
PICPGYRWMCLRRFI	S	67–81	[144]	DRB1*08, DRB1*11, DRB1*13	Predicted	ELISpot; Proliferation assay
GWSPQAQGVLTT	S	76–87	[145]	DRB1*0405	Predicted	ELISpot; Proliferation assay
MQWNSTTFHQTLQDPRVRGL	S	109–134	[47]	DRB1*01	Predicted	Immunization of mice; Proliferation assay; ELISpot
TTFHQTLQDPRVRGL	S	114–128	[47]	DRB1*01	Predicted	Immunization of mice; Proliferation assay; ELISpot
MQWNSTAFHQTLQDP	S	109–123	[146]	DRB1*02	Predicted	Proliferation assay; Cytotoxicity assay
STLPETTVVRRRGRSPRRRT	S	141–160	[139]	DRB1*13	overlapping	Proliferation assay
WASVRFSWLSLL	S	165–176	[147]	DRB1*11, DRB1*14	Predicted	CTL assay; Proliferation assay
VPFVQWFVGLSPTVW	S	177–191	[144]	DRB1*11	Predicted	ELISpot; Proliferation assay
QAGFFLLTRILTIPQS	S	179–194	[47]	DRB1*01	Predicted	Immunization of mice; Proliferation assay; ELISpot
WLSVIWMMWYWGPSL	S	191–205	[136]	DRB1*1202	overlapping	ICS
TSLNFLGGTTVCLGQ	S	200–214	[47]	DRB1*01	Predicted	Immunization of mice; Proliferation assay; ELISpot
GPSLYSIVSPFIPLL	S	202–216	[144]	DRB1*07	Predicted	ELISpot; Proliferation assay
LLPIFFCLWVYI	S	215–226	[147]	DRB1*07, DRB1*08, DRB1*14	Predicted	CTL assay; Proliferation assay
PICPGYRWMCLRRFIIFL	S	241–258	[148]	DRB1*0201	overlapping	Tetramer staining
FLLVLLDYQGMLP	S	256–268	[54]	DP4	Predicted	Immunization of mice; Proliferation assay; ELISpot
WEWASARFSWLSL	S	326–338	[54]	DP4	Predicted	Immunization of mice; Proliferation assay; ELISpot
WLSLLVPFVQWFVGL	S	335–349	[149]	DRB1*0101	Predicted	Immunization of mice; Pentamer staining; ELISpot; ICS; Cytotoxicity assay
SLLVPFVQWFVGLSPTVWLSV	S	337–357	[47]	DRB1*01	Predicted	Immunization of mice; Proliferation assay; ELISpot
SVRFSWLSLLVPFVQWF	S	343–357	[148]	DRB1*0201	overlapping	Tetramer staining
VGLSPTVWLSVI	S	347–358	[54]	DP4	Predicted	Immunization of mice; Proliferation assay; ELISpot
GLSPTVWLSVIW	S	348–359	[149]	DRB1*0101	Predicted	Immunization of mice; Pentamer staining; ELISpot; ICS; Cytotoxicity assay
TVWLSVIWMMWYW	S	352–364	[54]	DP4	Predicted	Immunization of mice; Proliferation assay; ELISpot

## 5. Conclusions

Here, we have taken an effort to present a reliable and updated T cell epitope repertoire of HBV. We summarized the statistics of 205 unique CD8^+^ T cell epitopes and 79 unique CD4^+^ T cell epitopes that have been experimentally validated and reported during the past 33 years, corresponding restricting HLA-molecule, and the methods to screen candidate epitopes and validate candidate epitopes. We hope that this review will be used as a tool for the design and development of therapeutic vaccines and T cell detection kits for HBV-infected patients.

## Data Availability

Not applicable.

## Abbreviations

HBV: Hepatitis B virus; HLA: human leukocyte antigen; LC: liver cirrhosis; HCC: hepatocellular carcinoma; CTL: cytotoxic T lymphocyte; ORF: open reading frame; cccDNA: covalently closed circular DNA; OLPs: overlapping peptides; PBMCs: peripheral blood mononuclear cells; IFN-γ ELISpot: IFN-γ enzyme-linked immunospot; ICS: intracellular cytokine staining.
